# Comparison of the outcomes between laparoscopic surgery and conventional open surgery in treating patients with stage II endometrial carcinoma: A protocol for systematic review and meta-analysis

**DOI:** 10.1097/MD.0000000000027148

**Published:** 2021-09-24

**Authors:** Ya Liu, Yunfeng Jin, Yuemei Lu, Xiaorong Zhong, Hong Lu

**Affiliations:** a Department of Obstetrics and Gynecology, Hai’an Hospital Affiliated to Nantong University, Nantong, Jiangsu, China; b Department of Obstetrics and Gynecology, Affiliated Hospital of Nantong University, Nantong, Jiangsu, China.

**Keywords:** conventional open surgery, laparoscopic surgery, meta-analysis, stage II endometrial carcinoma

## Abstract

**Background:**

Endometrial carcinoma is a prevalent form of cancer. In fact, its incidence ranks fourth among European and North American females. Moreover, it is the most common gynecological malignant disease. Laparotomy, bilateral salpingo-oophorectomy, total abdominal hysterectomy, etc were common methods adopted in conventional open surgery. Recent developments in laparoscopic surgery (LPS) has made it more effective. The present study aims to compare the outcomes between LPS and a conventional open surgical procedure to treat stage II endometrial carcinoma patients.

**Methods:**

A comprehensive search will be conducted on Cochrane library, PubMed, Web of Science, EMBASE, and China National of Knowledge Infrastructure to collect LPS and conventional open surgery in treating stage II endometrial carcinoma. The search will consider all articles published since the inception of the databases till July 2021. A pair of scholars will perform independent screening of the literature and extracted data to evaluate the bias risk in the selected studies. Afterwards, RevMan5.3 software will be used to conduct a meta-analysis.

**Conclusion:**

This study will conduct a meta-analysis to compare the clinical efficacy of LPS and conventional open surgery in the treatment of stage II endometrial carcinoma.

## 1 Introduction

Endometrial carcinoma is the fourth most prevalent form of cancer in North European and American females. It is also the most common gynecological malignant disease.^[1–4]^ In fact, endometrial carcinoma is primarily prevalent in postmenopausal women, and a majority of patients are women aged 50 years and above.^[5]^ However, due to lifestyle changes in people, the prevalence of metabolic disease rises, the incidence of endometrial cancer is significantly increasing globally among the youth.^[6]^ Surgical treatment is the first line of treatment for endometrial cancer and vaginal surgery. In addition, robotic surgery (RS), laparoscopic surgery (LPS), and laparotomy (LT) are effective for treating endometrial cancer. Previously, a randomized controlled trial exhibited that those who received LPS for endometrial cancer possessed better short-term clinical prognosis than patients who received LT.^[7]^ LT lessens the incidence of surgical complications in overweight and elderly females, which includes blood loss, wound infection, and intestinal obstruction.^[8]^ In recent years, there have been ground-breaking advancements in minimally invasive operating techniques, such as RS and laparoscopic. Still, the benefits of adopting RS over LPS and LT to treat endometrial cancer is yet to be established.^[9]^ Thus, the present meta-analysis aims to conduct a comparative analysis on the results of various surgical methods to assess the benefits of LPS in stage II endometrial cancer.

## 2 Materials and methods

### 2.1 Study search

A comprehensive search will be conducted on Cochrane library, PubMed, Web of Science, EMBASE, and China National of Knowledge Infrastructure to collect LPS and conventional open surgery in treating stage II endometrial carcinoma. The search will consider all articles published since the inception of the databases till July 2021. Numerous combinations of the keywords listed below will be used: “endometrial∗”; “neoplasia”; “carcinoma”; “tumor”; “tumour”; “cancer”; “surgery”; “malignancy”; “open”; “laparotomy”; “minimally invasive”. Reviewing the studies will also include the abstracts of all retrieved references from suitable studies.

### 2.2 Inclusion and exclusion criteria

The inclusion criteria are as follows:

(1) Randomized controlled experiments or non-randomized controlled experiments;
(2) Patients who were diagnosed with stage II endometrial cancer in accordance with the Federation International of Gynecology and Obstetrics^[10]^;
(3) Intervention measures include the comparison between RS (treatment group) and LPS or LT (control group) in treating endometrial cancer;
(4) Evaluation data include time taken for surgery, intra-operative blood loss, postoperative hospital stays, number of blood transfusions, complication rate, rate of readmission, complete rate of survival, disease-free survival rate, and number of lymph node resections;
(5) For literature published by the same author or institution, select the higher quality literature for statistics;
(6) English or Chinese literature;

The exclusion criteria are as follows:

(1) Letters, reviews, conferences, and case reports;
(2) The literature does not outline a comparative analysis of the therapeutic effects of RS and overall survival in endometrial cancer;
(3) Unable to extract data or obtain full-text documents;
(4) Repeatedly published documents by the same author or institution.

### 2.3 Outcome measures

The following perioperative outcomes will be used:

(1) Basic information: First author, publication time, research area, number of cases, age, body mass index, and pathological stage;
(2) Analytical indicators: Overall survival, recurrence free survival, estimated blood loss, operation time, intra-surgical problems, blood transfusion, postsurgical issues, total lymph nodes harvested, conversion to LT, the quantity of para-aortic lymph nodes harvested, the quantity of pelvic lymph nodes harvested, and hospitalized period/delayed discharge.

### 2.4 Data extraction and quality assessment

In accordance with the above inclusion criteria and exclusion criteria, the literature obtained by the search will be independently screened by 2 researchers. First, the researchers will read the titles and abstracts of all potential articles, and if it satisfies the requirements, the complete text will be scrutinized further to decide whether it is suitable for inclusion in the final selection. In the event of any disagreement, the 2 parties will discuss and resolve it; if it still cannot be resolved, a third party will be sought.

Perform literature quality evaluation; if it is a random control experiment, use the improved Jadad scale. If the research method is a retrospective study, the NOS evaluation scale will be used.^[11]^

### 2.5 Statistical analysis

Review Manager 5.3 will be used for the present meta-analysis. For dichotomous variables, the odds ratio will be calculated for analysis. For continuous variables with consistent measurement units, calculate the weighted mean difference, and calculate the standardized mean difference for continuous variables with inconsistent measurement units. The results were computed with 95% confidence interval, and *P* < .05 indicated that the difference was statistically significant. The *I*^2^ value is used to indicate the heterogeneity between the data. When *I*^2^ ≤ 50%, there is no obvious heterogeneity between the data, in which case the fixed-effects model will be adopted; when *I*^2^ > 50%, there is heterogeneity among the data, in which case the random-effects model will be adopted. A funnel chart is used to detect any possible publication bias.

## 3 Discussion

In traditional open surgery, the uterus is removed through open surgery. Intuitive surgery is performed under direct vision, and pelvic and para-aortic lymph nodes are dissected according to the specific conditions of the patient to achieve the effect of clearing the lesion. However, after open surgery, infection and peripheral organ damage are more likely to have a significant impact on the prognosis of patients. Recently, laparoscopic technology has developed to a great extent. Although there were certain controversies when it was first applied to the clinic, it has been improved with the improvement of surgical procedures and surgical instruments. It has become mature and has gradually gained recognition within the medical profession. Moreover, LPS has a wider and clearer field of view than before, which is beneficial for doctors to observe the patient's lesions, surrounding tissues, and organs, lymph node metastasis and perform electrosurgical resection, which greatly reduces the amount of bleeding. This article will further understand the therapeutic effect of LPS versus conventional open surgery through meta-analysis.

## Author contributions

**Conceptualization:** Ya Liu, Yunfeng Jin, Yuemei Lu, Hong Lu.

**Data curation:** Ya Liu, Yunfeng Jin.

**Formal analysis:** Ya Liu, Yunfeng Jin, Xiaorong Zhong.

**Funding acquisition:** Ya Liu, Xiaorong Zhong, Hong Lu.

**Investigation:** Ya Liu, Yunfeng Jin, Yuemei Lu.

**Methodology:** Ya Liu, Yunfeng Jin, Xiaorong Zhong, Hong Lu.

**Project administration:** Xiaorong Zhong.

**Resources:** Ya Liu, Yuemei Lu, Hong Lu.

**Software:** Yunfeng Jin, Yuemei Lu, Xiaorong Zhong, Hong Lu.

**Validation:** Ya Liu, Yunfeng Jin, Yuemei Lu, Xiaorong Zhong.

**Visualization:** Yunfeng Jin, Xiaorong Zhong, Hong Lu.

**Writing – original draft:** Ya Liu, Yunfeng Jin.

**Writing – review & editing:** Xiaorong Zhong, Hong Lu.
